# The effect of morbid obesity (BMI ≥ 35 kg/m^2^) on functional outcome and complication rate following unicompartmental knee arthroplasty: a case-control study

**DOI:** 10.1186/s13018-019-1316-5

**Published:** 2019-08-22

**Authors:** Ayşe Esin Polat, Barış Polat, Tahsin Gürpınar, Engin Çarkçı, Olcay Güler

**Affiliations:** 1 Department of Orthopaedics and Traumatology, Dr. Akçiçek State Hospital, 99300 Kyrenia, Cyprus; 2 Department of Orthopaedics and Traumatology, University of Kyrenia, Faculty of Medicine, 99138 Kyrenia, Cyprus; 3 Department of Orthopedics and Traumatology, Istanbul Training and Resarch Hospital, 34098, Fatih/Istanbul, Istanbul, Turkey; 4 Department of Orthopedics and Traumatology, Medicalpark Bahçelievler Hospital, 34180 Istanbul, Turkey

**Keywords:** Unicompartmental arthroplasty, Obesity, Knee anteromedial osteoarthritis

## Abstract

**Background:**

The aim of this study was to evaluate and compare the functional outcomes and complication rates of patients in short-term and midterm follow-up period when medial unicompartmental knee arthroplasty (UKA)-applied patients were grouped according to BMI values.

**Methods:**

One hundred four patients (mean age 60.2 ± 7.4 (range, 49–80)) to whom medial UKA was applied between 2011 to 2016 with a minimum of 2 years follow-up were grouped as normal and overweight (less than 30 kg/m^2^), obese (30–34.9 kg/m^2^) and morbidly obese (BMI ≥ 35 kg/m^2^) according to their BMI. The postoperative Knee Society Scores (KSS), functional Knee Society Scores (fKSS), Oxford Knee Scores (OKS), visual analogue scale (VAS) and range of motion (ROM) results and complication rate of these groups were compared statistically. The implant positioning of the patients requiring revision was analysed according to the Oxford radiological criteria.

**Results:**

The average BMI of 104 patients was 34.4 (range, 22–56.9). Twenty-six (25%) of these were normal or overweight, 40 (38.5%) were obese and 38 (36.5%) were morbidly obese. However, in these BMI groups, there was no significant difference between the preoperative VAS, postoperative VAS and VAS score changes among these three groups (*p* > 0.05). The postop KSS, *f* KSS and OKS were significantly poorer in the morbidly obese group by 75.2, 70.5 and 33.1, respectively. Furthermore, amount of ROM changes (4.2°) were significantly poorer in the morbidly obese group (*p* < 0.05). Complications including eminence fractures, insert dislocations, tibial component collapses and superficial infections developed in 10 patients (9.6%). Six of them (60%) were morbidly obese, and four of them (40%) were obese. Furthermore, 11 (10.6%) of the patients required revision. Eight (72.7%) of the patients were morbidly obese, and three (27.3%) of them were obese.

**Conclusions:**

We concluded that morbid obesity is an independent risk factor for functional outcomes and implant survival after UKA. However, it is possible to obtain excellent results for obese and overweight patients with good planning and correct surgical technique. Morbid obese patients should be preoperatively informed about poor functional outcome and high complication rate. Treatment of morbid obesity before UKA surgery may be a good option.

## Background

One of the most common joint problems experienced around the world is osteoarthritis [1]. The prolongation of life expectancy, the increase in obesity, and the spread of immobile lifestyles are increasing the frequency of knee osteoarthritis [2]. Obesity is often associated with knee osteoarthritis. This is a group of patients whom orthopaedic surgeons often encounter and treat. When a body mass index (BMI) between 25 and 29.9 is considered overweight and 30 to 34.9 is obese, it has been shown that the risk of osteoarthritis of the knee increases almost fivefold (4.78) in obese men and almost fourfold (3.87) in obese women; however, an increase of 1.69-fold in overweight men and 1.89-fold in overweight women was observed [3].

Osteoarthritis usually starts from the medial compartment (80–90%) and largely remains unicompartmental [4]. There are different types of surgical treatment for single-compartment osteoarthritis, including total knee arthroplasty (TKA), high tibial osteotomy (HTO) or unicompartmental knee arthroplasty (UKA). Although the prevalence of revision frequency is higher than that of TKA [5] and the possibility of the opposite compartment going to arthrosis limits the preference of surgeons regarding UKA, faster recovery, less hospitalization time and reduced costs lead many surgeons to view it as an alternative surgical method instead of TKA [5, 6]. Fatal complications such as infections, thromboembolism and amputation are rarely seen in UKA compared to TKA [7]. In addition, the minimally invasive incision reduces postoperative blood loss and pain, while better functional results, as well as rapid and early rehabilitation, are the other advantages of UKA [5, 6].

In recent studies, UKA has been considered the preferred replacement method over TKA and HTO in the presence of single-compartment arthrosis [5, 6]. In the recent past, UKA accounted for 8% of all arthroplasty practices, although this rate is steadily increasing at a rate of 30% per year in the USA [8]. In some series, this rate has reached up to 50% of all replacement knees [6].

In order to achieve successful outcomes, correct patient selection is one of the most important criteria following UKA. However, there are still some contraversion for indication criteria of UKA, including obesity. While BMI is a restriction in traditional indications, in recent decades, this BMI-based restriction has not been possible because of the increasing obesity pandemic that has been observed throughout the world. Although obesity is believed to reduce the functional results and implant survival in UKA [9–13], successful results have been shown for obese patients in recent studies [14–18]. The effect of BMI on functional outcomes and complication rate after UKA still remains controversial, despite the plethora of studies. Although we had a satisfactory result in obese patients in our case series, we wanted to evaluate our patients retrospectively and planned this study after having observed poor functional outcome and early failure in morbid obese patients.

The aim of this study was to evaluate and compare the functional outcomes and complication rates of patients in short-term and midterm follow-up period when medial UKA-applied patients were grouped according to BMI values.

## Methods

After receiving ethical council approval (Reference No: 47, 07/06/2016), the medical records for all patients who underwent surgical treatment for single-compartment arthroplasty between 2011 and 2016 with a minimum 2 years follow-up were examined retrospectively. All operations were performed by a same surgeon (OG) or under his control in a single center. Application of Oxford Phase 3 medial unicompartmental cemented mobile bearing knee implants (Oxford Partial Knee, Biomet Orthopaedics, Bridgend, UK) were performed minimally invasively by the medial parapatellar approach.

Patients who had anteromedial knee arthritis except for inflammatory aetiologies were included. The inclusion criteria for UKA were determined as having an intact cruciate ligament, full-thickness lateral cartilage, flexion contracture less than 15° and fully correctable intra-articular varus deformity [19]. No restrictions were applied according to bodyweight or BMI. Fifty-two patients who did not complete the 2-year follow-up period, 8 patients who lost from the postoperative follow-up, 1 patient who underwent lateral UKA and 2 patients who underwent arthrosurface hemicap procedure were excluded from this study. The remaining 104 patients were grouped according to BMI: normal and overweight group (less than 30 kg/m^2^), obese group (30–34.9 kg/m^2^) and morbidly obese group (BMI 35 and more than 35 kg/m^2^).

All patients were evaluated preoperatively with bilateral orthoroentgenograms, weight-bearing anteroposterior and flexed lateral knee X-rays and patella tangential X-rays. In addition, magnetic resonance imagining (MRI) was performed preoperatively. Postoperatively, bilateral orthoroentgenograms and weight-bearing anteroposterior and flexed lateral knee X-rays were taken.

The visual analogue scale (VAS) score, Oxford Knee Score (OKS), Knee Society Score (KSS) and functional Knee Society Score (fKSS) were used for functionally evaluating the patients, and the range of motion (ROM) was recorded for each patient pre- and postoperatively. The demographic features, functional results, preoperative and postoperative VAS and ROM and complication rate of the three subgroups of normal and overweight, obese and morbidly obese were compared. The implant positioning of the patients requiring revision was analysed according to the Oxford radiological criteria. The presence of femoral component varus or valgus of more than 10°, femoral component flexion or extension of more than 5°, tibial plateau angle varus or valgus of more than 10° and posterior tibial slope of more than 7° or less than 5° according to the normal value was evaluated as implant malposition.

In the descriptive statistics of the data, mean, standard deviation, median lowest, highest, frequency and ratio values were used. The distribution of the variables was measured with the Kolmogorov-Smirnov test. In the analysis of the quantitative independent data, the Kruskal-Wallis and Mann-Whitney *U* tests were used. The Wilcoxon signed-rank test was used for the analysis of the dependent quantitative data. The chi-square test was used for the analysis of qualitative independent data, and the Fischer test was used when the chi-square test conditions were not provided. The SPSS 22.0 programme was used in the analysis. A statistical significance level of alpha was accepted as *p* < 0.05.

## Results

In total, 86 patients (82.7%) were female and 18 (17.3%) were male. The mean age of the patients was 60 (range, 49–80). The left knee of 46 patients and the right knee of 58 patients underwent UKA. The mean follow-up period was 46 (range, 24–72) months. The average BMI was 34.4 (range, 22–56.9). A total of 26 (25%) of the patients were either normal weight or overweight, 40 (38.5%) were obese and 38 (36.5%) were morbidly obese. The age, gender and side distribution of the patients in the normal-overweight, obese and morbidly obese group did not differ significantly (*p* > 0.05).

For all of these groups, the average KSS, *f* KSS and OKS improved from 43.2, 34.7 and 12.7 to 85.7, 82.0 and 37.8, respectively. Additionally, the range of knee motion improved from 111.5 to 122.8 and the average VAS score decreased from 9.0 to 2.6 (*p* < 0.05) (Table 1).

**Table 1:** All patient demographics and functional outcome

		Min–Max	Median	Mean ± s.d./*n*-%
Age		49.0–80.0	60.0	60.2 ± 7.4
Sex	Female			86 82.7%
	Male			18 17.3%
Side	Right			58 55.8%
	Left			46 44.2%
Follow-up (month)		24.0–72.0	43.5	46.0 ± 14.6
BMI		22.0–56.9	33.4	34.4 ± 6.5
VAS	Preop	6.0–10.0	9.0	1.1 ± 9.0
	Postop	0.0–10.0	2.6	3.0 ± 2.0
KSS	Preop	17.0–69.0	43.2	9.6 ± 44.0
	Postop	31.0–100.0	85.7	19.8 ± 95.0
fKSS	Preop	0.0–90.0	34.7	18.9 ± 35.0
	Postop	0.0–100.0	82.0	24.3 ± 90.0
OKS	Preop	0.0–32.0	12.7	7.7 ± 11.5
	Postop	7.0–48.0	37.8	10.3 ± 41.0
ROM	Preop	80.0–130.0	111.5	12.4 ± 115.0
	Postop	35.0–135.0	122.8	16.8 ± 130.0
Complication				10 9.6%
Revision				11 10.6%

In these BMI-based groups, there were statistically significant differences according to KSS, *f* KSS, OKS and functional ROM. There was no significant difference between preoperative VAS, postoperative VAS and VAS score changes in the normal-overweight, obese and morbidly obese patients (*p* > 0.05). There was no significant difference between preoperative KSS, *f* KSS and OKS in normal-overweight, obese and morbidly obese patients (*p* > 0.05). Postoperative KSS, *f* KSS and OKS were significantly lower in the morbidly obese group in comparison to the normal-overweight and obese group by 75.2, 70.5 and 33.1, respectively (*p* < 0.05). Postoperative KSS, *f* KSS and OKS changes were significantly lower in the morbidly obese group than in the normal-overweight and obese group (*p* < 0.05). In all groups, the postoperative ROM scores increased significantly (*p* < 0.05) compared to the preop period (*p* < 0.05). Postoperative functional ROM scores and amount of ROM changes (4.2°) were significantly lower in the morbidly obese group compared to the normal-overweight (11.9°) and obese group (17.6°) (*p* < 0.05) (Table 2).

**Table 2:** Comparison of patient demographics, VAS, KSS, *f* KSS, OKS and ROM in BMI groups

		Normal and Overweight			Obese			Morbidly Obese			
		Mean ± s.d./*n*-%		Median	Mean ± s.d./*n*-%		Median	Mean ± s.d./*n*-%		Median	
Age		61.5 ± 7.3		61.0	60.5 ± 7.7		60.0	59.0 ± 7.1		58.0	0.374^K^
Sex	Female	22	84.6%		32	80.0%		32	84.2%		0.847^x2^
	Male	4	15.4%		8	20.0%		6	15.8%		
Side	Right	12	46.2%		24	60.0%		22	57.9%		0.513^x2^
	Left	14	53.8%		16	40.0%		16	42.1%		
Follow-up		42.7 ± 14.1ǂ		36.0	40.6 ± 13.5ǂ		40.0	53.9 ± 12.7		55.0	0.000^K^
BMI		27.3 ± 2.3		28.0	32.7 ± 1.5		33.1	40.9 ± 5.6		39.0	
VAS											
Preoperative		8.9 ± 0.9		9.0	9.2 ± 1.0		10.0	8.8 ± 1.2		9.0	0.232^K^
Postoperative		1.6 ± 1.9		1.0	2.3 ± 2.4		2.0	3.7 ± 3.8		2.0	0.099^K^
Change		− 7.3 ± 1.9		− 7.0	− 6.9 ± 2.8		− 7.0	− 5.2 ± 4.2		− 6.0	0.195^K^
Group exchange *p*		0.000^w^			0.000^w^			0.000^w^			
KSS											
Preoperative		44.0 ± 4.3		44.0	42.1 ± 11.8		43.5	43.9 ± 9.8		47.0	0.158^K^
Postoperative		96.3 ± 6.1*ǂ		100.0	88.8 ± 10.8		93.5	75.2 ± 27.2		82.0	0.001^K^
Change		52.3 ± 6.6ǂ		55.0	46.7 ± 14.6ǂ		48.5	31.2 ± 28.3		45.0	0.005^K^
Group exchange *p*		0.000^w^			0.000^w^			0.000^w^			
fKSS		0.0									
Preoperative		35.8 ± 22.2		50.0	32.3 ± 21.2		35.0	36.6 ± 13.5		35.0	0.245^K^
Postoperative		90.0 ± 12.6ǂ		90.0	87.8 ± 12.4ǂ		90.0	70.5 ± 34.0		80.0	0.013^K^
Change		54.2 ± 18.4ǂ		50.0	55.5 ± 27.7ǂ		60.0	33.9 ± 35.5		45.0	0.015^K^
Group exchange *p*		0.000^w^			0.000^w^			0.000^w^			
OKS											
Preoperative		11.4 ± 7.8		11.0	11.4 ± 8.0		10.0	15.1 ± 7.0		16.0	0.071^K^
Postoperative		42.5 ± 4.7ǂ		43.0	39.3 ± 7.2ǂ		40.5	33.1 ± 13.6		40.0	0.011^K^
Change		31.1 ± 8.6ǂ		30.0	27.9 ± 10.3ǂ		30.0	18.0 ± 16.8		25.0	0.003^K^
Group exchange *p*		0.000^w^			0.000^w^			0.000^w^			
ROM											
Preoperative		116.3 ± 12.0*		120.0	106.9 ± 11.2		110.0	113.2 ± 12.5*		115.0	0.002^K^
Postoperative		128.3 ± 19.3*ǂ		130.0	124.5 ± 11.8		130.0	117.4 ± 18.3		120.0	0.002^K^
Change		11.9 ± 17.7ǂ		12.5	17.6 ± 15.6ǂ		20.0	4.2 ± 16.0		5.0	0.001^K^
Group exchange *p*		0.000^w^			0.000^w^			0.026^w^			

^K^ Kruskal-Wallis (Mann-Whitney *U* test)/^w^Wilcoxon test/^X2^chi-square test (Fischer test)

*Difference with the obese group *p* < 0.05/ǂcompared with the morbid obese group *p* < 0.05

While 100% of the normal and overweight patients reached excellent postoperative KSS, only 80% of the obese and 57.9% of the morbidly obese patients reached excellent results. Also, good and excellent functional KSS were reached by 92.3% of the normal and overweight patients, 95% of the obese patients and 73.7% of the morbidly obese patients postoperatively. Additionally, postoperative OKS were good and excellent for 100% of the normal and overweight patients, 95% of the obese patients and 68.4% of the morbidly obese patients (Table 3).

**Table 3:** Postop KSS, *f* KSS and OKS result distribution of BMI groups

		BMI					
		Normal-overweight		Obese		Morbidly obese	
		*n*	%	*n*	%	*n*	%
Post KSS	Excellent	26	100	32	80	22	57.9
	Good	0	0	6	15	4	10.5
	Fair	0	0	2	5	2	5.3
	Poor	0	0	0	0	10	26.3
Post *f* KSS	Excellent	22	84.6	30	75	24	63.2
	Good	2	7.7	8	20	4	10.5
	Fair	2	7.7	2	5	0	0
	Poor	0	0	0	0	10	26.3
Post OKS	Excellent	24	92.3	35	87.5	22	57.9
	Good	2	7.7	3	7.5	4	10.5
	Fair	0	0	1	2.5	2	5.3
	Poor	0	0	1	2.5	10	26.3

Complications developed in ten patients (9.6%). Three of the complications were intraoperative eminence fractures, two of them were insert dislocations, four of the complications were tibial component collapse and superficial infection developed in one of them, which healed with antibiotic therapy. Furthermore, six of the ten patients (60%) who developed complications were morbidly obese and four of them (40%) were obese. In this study, complications were observed in 6 (16%) of 38 morbidly obese patients and 4 (10%) of 40 obese patients. No complication was observed in 26 normal or overweight patients.

**Fig. 1:**
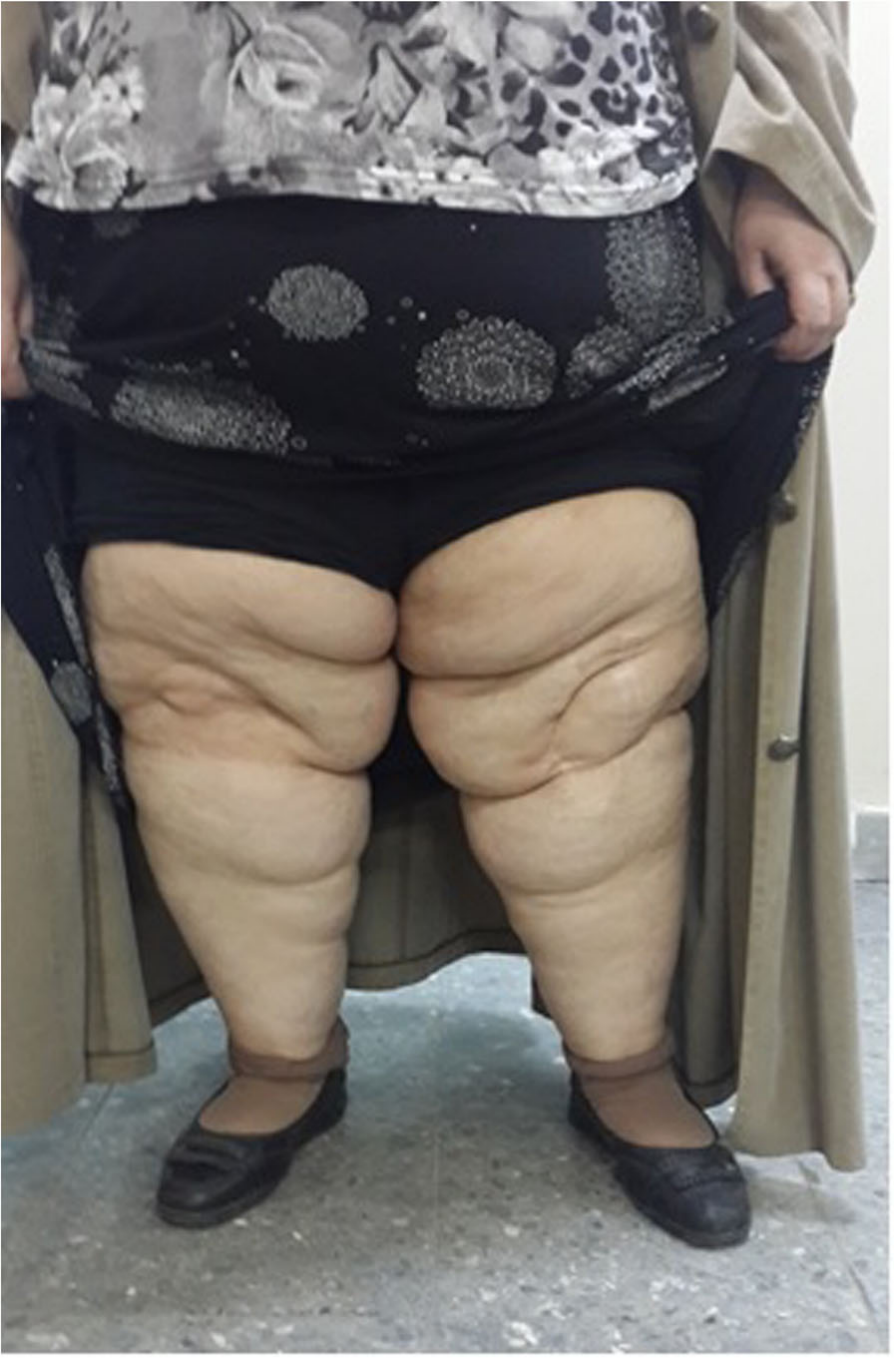
53-year-old 56.9 BMI patient clinical view

**Fig. 2:**
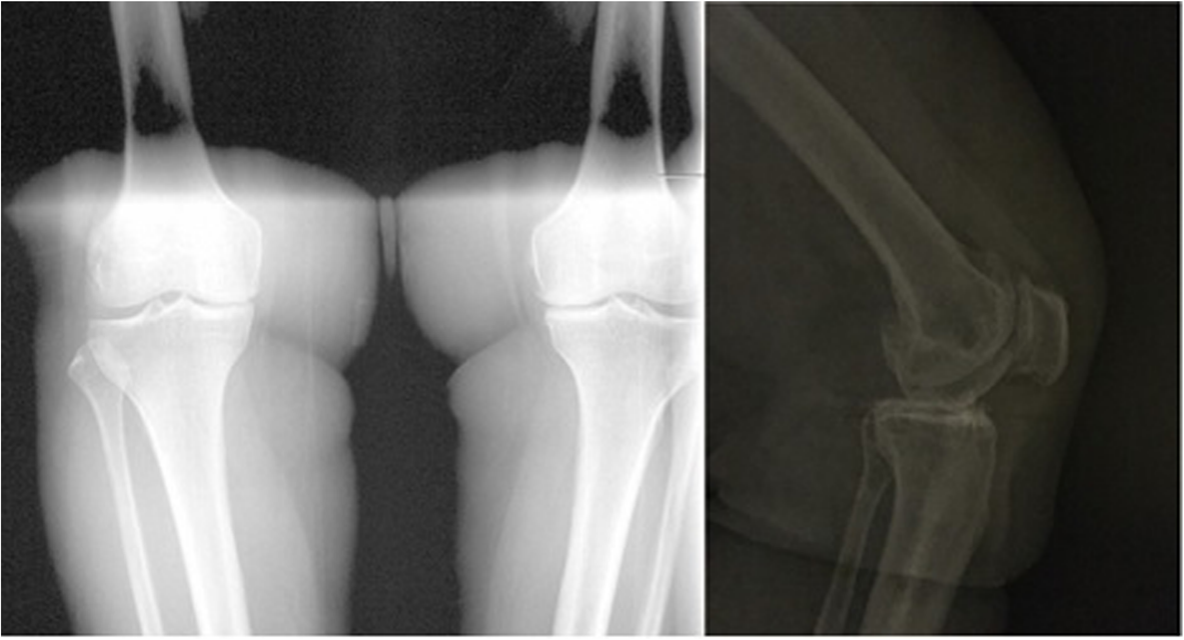
Preoperative AP and lateral X-ray of the same patient

**Fig. 3:**
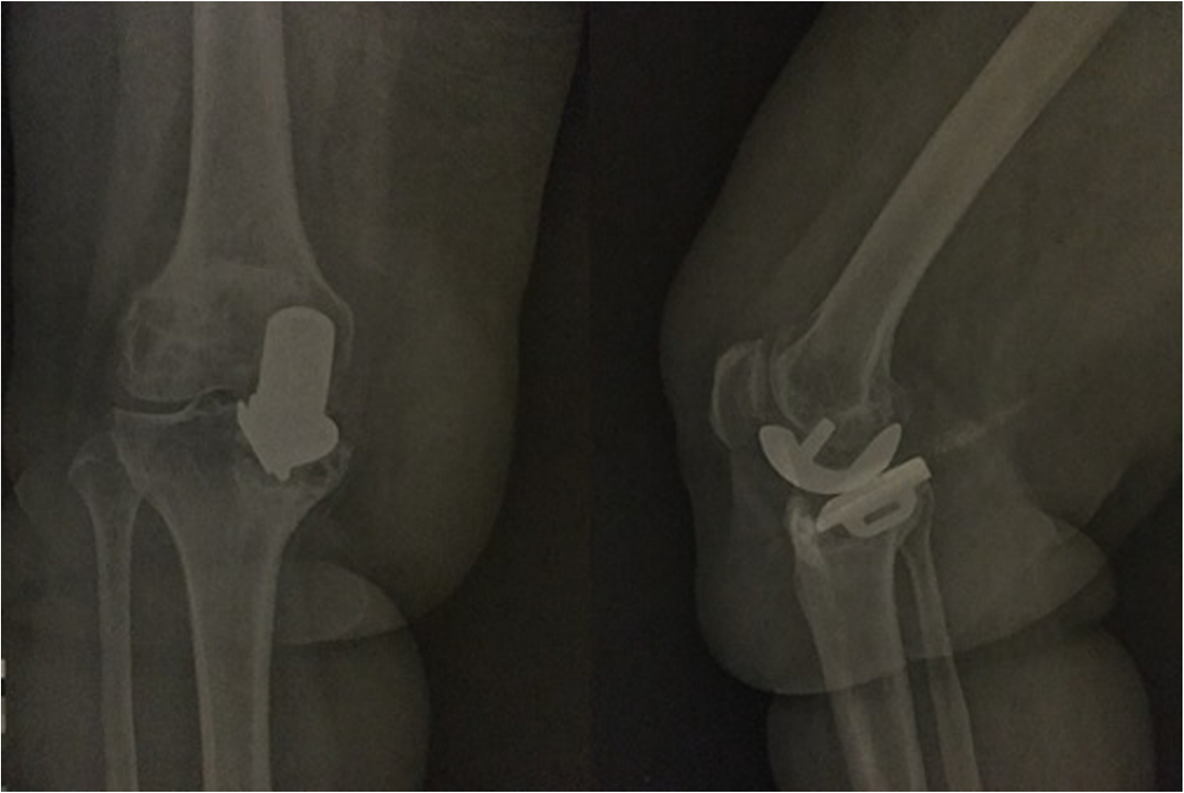
Postoperative 12 months AP and lateral X-ray of the same patient. UKA of this patient was revised at 12 months with constraint THA

Eleven (10.6%) of the patients required revision. A total of 8 (72.7%) of these patients were morbidly obese and 3 (27.3%) of them were obese. Four morbidly obese patients underwent revision in the first 12 months. Thirty-eight (36.5%) of the total of 104 knees were in morbidly obese patients and 8 (21.1%) of these patients underwent revision. Furthermore, 5 of these 8 patients had one or more implant replacement errors and 3 of them had no implant replacement errors by the time of the UKA replacement. Three of the 8 morbidly obese patients had no other cause for revision except BMI (Figs. 1, 2, and 3). Two obese patients who needed revision also had implant replacement errors, and one of them had no implant replacement errors (Table 4). Also, there is a significant difference in the mean BMI between patient groups who required surgery and those who did not. While the mean BMI of patients requiring revision was 41.7, the mean BMI for those not requiring revision was 33.5. Also, one of the morbidly obese patients who underwent revision again experienced implant loosening of TKA and revision was planned.

**Table 4:** Summary of revised patient findings

	BMI	Patient age	Comp. Malp	Complication	Revision reason	Revision time	Revision implant
1	38,7	53	PTS, TPA	Tibial component collapse	Tibial loosening	52 months	Constraint TKA
2	33	65	PTS	Eminence	Tibial+femoral loosening	45 months	Primer PCL substituting TKA
3	41.6	58	FCVV	Eminence fracture	Tibial loosening	7 months	Primer PCL retaining TKA
4	47	51	None	None	Tibial+femoral loosening	7 months	Primer PCL retaining TKA
5	56.9	53	None	Tibial component collapse	Tibial component collapse	12 months	Constraint TKA
6	46.9	56	PTS, TPA, FCVV	Insert dislocation	Tibial+femoral loosening	12 months	Primer PCL retaining TKA
7	38.1	53	PTS, TPA	None	Tibial loosening	14 months	Primer PCL retaining TKA
8	42.5	58	None	Tibial component collapse	Tibial component collapse	26 months	Primer PCL retaining TKA
9	33.2	58	FCVV, FCFE	None	Tibial+femoral loosening	33 months	Primer PCL substituting TKA
10	46.9	56	PTS, TPA, FCVV	None	Tibial+femoral loosening	16 months	Primer PCL substitutingTKA
11	34.7	80	None	Tibial component collapse	Tibial+femoral loosening	44 months	Constraint TKA

*PTS* posterior tibial slope, *TPA* tibia plateau angle, *FCVV* femoral component varus-valgus, *FCFE* femoral component flexion-extension, *PCL* posterior cruciate ligament

## Discussion

The popularity of UKA has increased as excellent functional outcomes have been obtained even in long-term follow-up, and it has significant advantages compared to TKA. However, the weight or BMI of patients undergoing UKA is still controversial in the literature. In 1989, obesity was determined to be a contraindication for UKA and Kozin et al. restricted their indication criteria to 82 kg [20]. While Deshmukh and Scott expanded this limit to 90 kg [21], Berend and Lambordi [19] did not include bodyweight in their indication criteria and Murray et al. showed that high BMI values such as 45–50 do not represent a restriction criteria in patients who undergo mobile insert UKA [18]. Excess weight increases the implant interface stress and may lead to early implant loosening. Although there are many studies supporting this conviction [9–13], there are other studies claiming the opposite [14–18]. In this study, no restriction was applied according to body weight or BMI. Therefore, without making any restrictions in terms of weight, most of the patients (75%) who reached the inclusion criteria for UKA surgery were obese (38.5%) or morbidly obese (36.5%).

Component malposition is believed to increase revision rates. In such a study, femorotibial angle, tibial plateau angle, posterior tibial slope and the height of the joint line were significantly different in patients with failure after UKA [22]. However, in three of eight morbidly obese patients who underwent revision surgery, component malposition was not detected in any patient according to the Oxford radiological criteria. In these three morbidly obese patients, although the component was implanted appropriately, revision was needed.

Another reason for revision knee replacement could be that the patients are younger and more active. Price et al. presented that the survival rate was 91% in patients under 60 years of age, while survival was 96% in patients over 60 years of age after 10 years of UKA [23]. The mean age of the all patients was 60.2 (range, 49–80), while patients who underwent revision surgery was 58.2 (range, 51–80). This situation shows that the mean age of the patient group requiring revision is close to the mean age of the total patient group. When we refute factors such as component positioning and age, we conclude that morbid obesity is an independent risk factor for functional outcomes and implant survival after UKA.

In a large-scale study conducted with 15,770 patients who underwent UKA, the major complication rate was 3.1 times higher and the revision rate 2.1-fold higher in patients with morbid obesity compared to non-obese patients [9]. Similarly, 40 patients with and without morbid obesity were treated with UKA with a minimum 2 years follow-up, and 5 revisions were needed. Five of these patients were also in the morbidly obese group [12]. In this study, of the ten patients who developed complications, six were morbidly obese and four were obese. Eight of the eleven patients who underwent revision surgery were morbidly obese and three were obese.

The limitation of this study is its retrospective design; the BMI of the patients at the operation time are taken as reference, and their last follow-up BMI values are not taken into consideration. Furthermore, a larger series is needed in terms of the number of patients and follow-up time.

## Conclusion

We recommend that morbid obesity is treated before surgical planning because it affects implant survival and functional outcomes. However, it is possible to obtain excellent results with good planning and correct surgical technique in obese and overweight patients. Morbid obese patients should be preoperatively informed about poor functional outcome and high complication rate. Treatment of morbid obesity before UKA surgery may be a good option.

## Data Availability

The data of this study were real and were performed in SPSS (Version 22.0). The statistical results of the data are presented in the main paper. All of the data are available in contact with the correspondence author.

## Abbreviations

BMI: Body mass index
fKSS: Functional Knee Society Scores
HTO: High tibial osteotomy
KSS: Knee Society Scores
MRI: Magnetic resonance imaging
OKS: Oxford Knee Scores
ROM: Range of motion
TKA: Total knee arthroplasty
UKA: Unicompartmental knee arthroplasty
VAS: Visual analogue scale
